# Lymphatic filariasis transmission on Mafia Islands, Tanzania: Evidence from xenomonitoring in mosquito vectors

**DOI:** 10.1371/journal.pntd.0005938

**Published:** 2017-10-06

**Authors:** Yahya A. Derua, Susan F. Rumisha, Bernard M. Batengana, Demetrius A. Max, Grades Stanley, William N. Kisinza, Leonard E. G. Mboera

**Affiliations:** 1 National Institute for Medical Research, Amani Research Centre, Muheza, Tanga, Tanzania; 2 National Institute for Medical Research, Headquarters, Dar es Salaam, Tanzania; Erasmus MC, NETHERLANDS

## Abstract

**Introduction:**

Lymphatic filariasis (LF) is a chronic nematode infection transmitted by mosquitoes and in sub-Saharan Africa it is caused by *Wuchereria bancrofti*. The disease was targeted for global elimination by 2020 using repeated community-wide mass drug administration (MDA) distributed in endemic areas. However, recently, there has been a growing recognition of the potential role of including vector control as a supplement to MDA to achieve elimination goal. This study was carried out to determine mosquito abundance and transmission of bancroftian filariasis on Mafia Islands in Tanzania as a prerequisite for a search for appropriate vector control methods to complement the ongoing MDA campaign.

**Methods:**

Mosquitoes were collected indoor and outdoor using Centre for Disease Control (CDC) light and gravid traps, respectively. Collected mosquitoes were identified based on their differential morphological features and *Anopheles gambiae* complex and *An*. *funestus* group were further identified to their respective sibling species by polymerase chain reaction (PCR). Filarial mosquito vectors were then examined for infection with *Wuchereria bancrofti* by microscopy and PCR technique.

**Results:**

Overall, a total of 35,534 filarial mosquito vectors were collected, of which *Anopheles gambiae* complex, *An*. *funestus* group and *Culex quinquefasciatus* Say accounted for 1.3, 0.5 and 98.2%, respectively. Based on PCR identification, *An*. *gambiae* sensu stricto (s.s) and *An*. *funestus* s.s sibling species accounted for 88.3% and 99.1% of the identified members of the *An*. *gambiae* complex and *An*. *funestus* group, respectively. A total of 7,936 mosquitoes were examined for infection with *W*. *bancrofti* by microscopy. The infection and infectivity rates were 0.25% and 0.08%, respectively. Using pool screen PCR technique, analysis of 324 mosquito pools (each with 25 mosquitoes) resulted to an estimated infection rate of 1.7%.

**Conclusion:**

The study has shown that *Cx*. *quinquefasciatus* is the dominant mosquito on Mafia Islands. By using mosquito infectivity as proxy to human infection, the study indicates that *W*. *bancrofti* transmission is still ongoing on Mafia Islands after more than a decade of control activities based on MDA.

## Author summary

Lymphatic filariasis is a chronic human disease caused by parasitic worms and transmitted by mosquitoes. The disease is targeted for elimination by 2020 through the treatment of the entire population at risk in endemic areas using a mass drug administration (MDA) strategy. After several years of MDA, there is now growing interest in including vector control as a supplement to MDA to achieve elimination goal. This study was carried out to determine mosquito abundance and transmission of lymphatic filariasis on Mafia Islands in Tanzania after nine rounds of MDA. Mosquitoes were collected indoor and outdoor using Centre for Disease Control (CDC) light and gravid traps, respectively. Filarial mosquito vectors were examined for infection with *Wuchereria bancrofti* by microscopy and PCR technique. A total of 35,534 filarial mosquito vectors were collected, of which *Anopheles gambiae* complex, *An*. *funestus* group and *Culex quinquefasciatus* Say accounted for 1.3, 0.5 and 98.2%, respectively. Using PCR, *An*. *gambiae* sensu stricto (s.s) and *An*. *funestus* s.s sibling species accounted for 88.3% and 99.1% of the identified members of the *An*. *gambiae* complex and *An*. *funestus* group, respectively. A total of 7,936 mosquitoes were examined for infection with *W*. *bancrofti* by microscopy. The infection and infectivity rates were 0.25% and 0.08%, respectively. Using PCR technique, of 324 mosquito pools (each with 25 mosquitoes) tested, 115 were found to be infected with at least a larval stage of *W*. *bancrofti*. The study concludes that *Cx*. *quinquefasciatus* is the dominant mosquito on Mafia Islands and that *W*. *bancrofti* transmission is still ongoing on Mafia Islands after a decade of control activities based on MDA.

## Introduction

Lymphatic filariasis (LF) is a chronic infection with serious physical, mental and socio- economic consequences to the affected individuals, and ranked as one the leading causes of long-term disability in the world [1, 2]. In Sub-Saharan Africa, LF is caused by the filarial nematode *Wuchereria bancrofti* and transmitted mainly by *Anopheles* and *Culex* mosquitoes [3]. Globally, it has been estimated that more than one billion people live in endemic areas and are at risk of infection, and more than one third of these are in Sub-Saharan Africa [4]. In Tanzania, it has been estimated that 34 million people are at risk of LF infection and about 6 million live with debilitating manifestations of the disease [5].

LF was considered eradicable and the World Health Organization (WHO) launched a Global Programme to Eliminate Lymphatic Filariasis (GPELF) by year 2020 [6]. The principal elimination strategy in endemic countries is based on yearly community-wide mass drug administration (MDA) with ivermectin or diethyl-carbamazine in combination with albendazole [6, 7]. The drugs mainly kill microfilariae and it is assumed that the reduction of microfilarial load in endemic communities will lead to reduction or even elimination of transmission [8]. Since the inception of GPELF, countries have initiated their local control programmes and encouraging reduction in disease prevalence as a result of MDA have been reported elsewhere [9, 10]. In Tanzania, MDA intervention was launched on Mafia Islands in the year 2000 and geographical coverage has been expanded in most of the endemic districts [5, 11].

Recently, there is growing recognition of the potential role of inclusion of vector control to achieve interruption of LF transmission in different epidemiological settings [3, 12]. In line with this assumption, studies have indicated that use of insecticide treated bed nets (ITNs) resulted in reduction in prevalence and transmission of LF [13–17]. However, insecticide based mosquito vector control interventions are threatened by development of insecticide resistance [18] and change in behaviour or shift of mosquito vectors species [19, 20]. Other studies have shown that *Cx*. *quinquefasciatus*, an important filarial vector is relatively tolerant to insecticides used for ITNs and IRS interventions [13, 21]. Thus, to expedite LF elimination efforts, novel control methods are needed to tackle the growing population of insecticide tolerant *Cx*. *quinquefasciatus* which is responsible for most of LF transmission in Tanzania as previously reported [19].

This study was carried out to determine mosquito abundance and transmission of bancroftian filariasis on Mafia Islands in Tanzania as a prerequisite for a search for appropriate vector control method to complement the ongoing MDA campaign. Xenomonitoring in filarial vectors has been considered as an integral component of monitoring the impact of MDA and has been reported to provide real time information on LF transmission [22, 23]. For mosquito surveys, both Centre for Disease Control (CDC) light and gravid traps have been found to be useful tools for collection of filarial mosquito vectors [24, 25]. Dissections of the vectors and molecular tests based on polymerase chain reaction (PCR) have proved useful in detection of *W*. *bancrofti* in mosquitoes [19, 26, 27]. The potential of PCR to screen large number of mosquitoes relatively quickly with high precision are requirements when infection rates in mosquitoes decrease after repeated MDA cycles. By using xenomonitoring as a proxy to human infection, this study reports *W*. *bancrofti* transmission on Mafia Islands 15 years after the launching of MDA campaign by the Tanzanian National Lymphatic Filariasis Elimination Programme.

## Methods

### Study site

**Fig 1:**
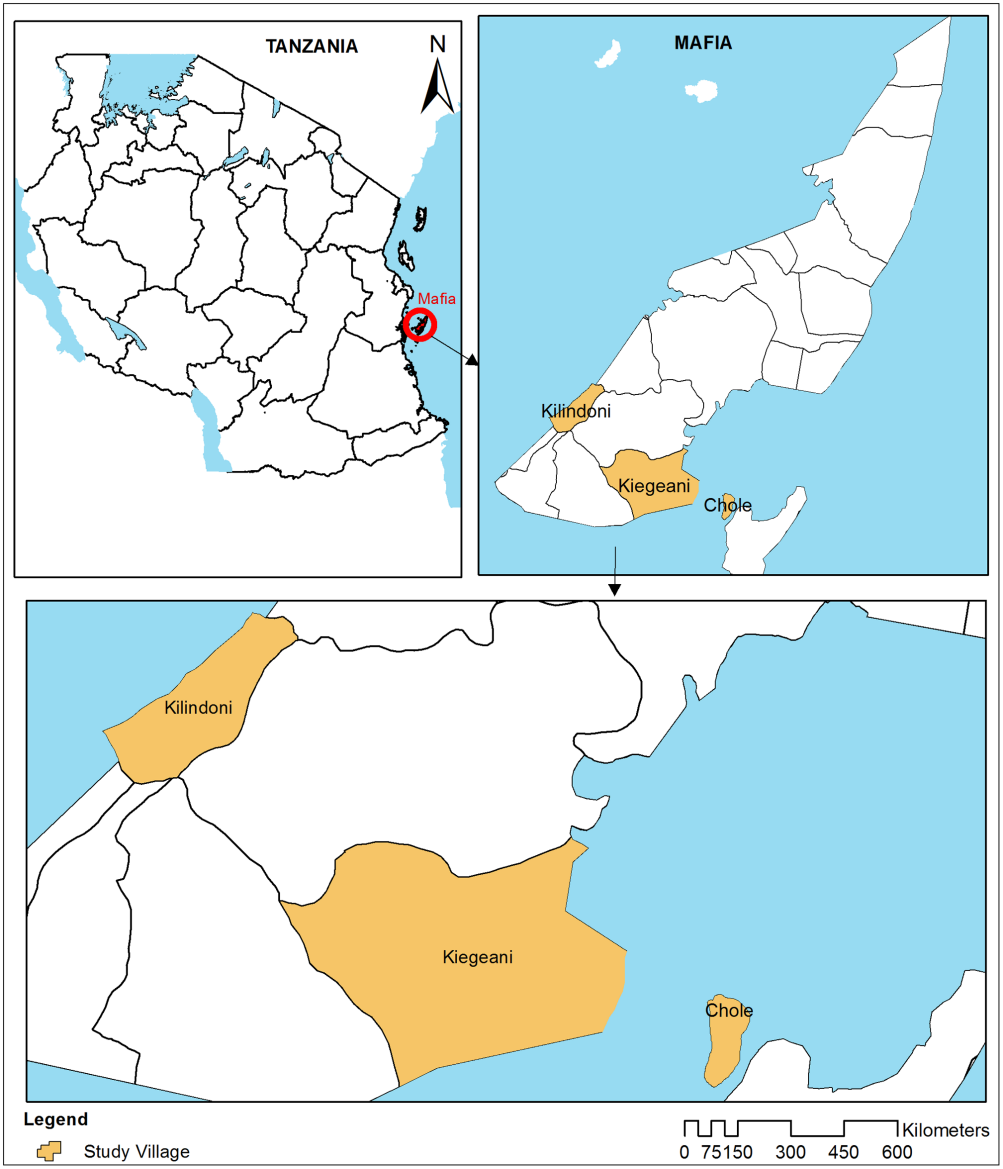
Mafia Islands in Tanzania showing location of the study villages.

The study was conducted on Mafia Islands (07°91554’S, 39°65529’E) in the Indian Ocean, off shore of the Pwani Region at about 195 km south-east of Dar es Salaam, Tanzania. A distance of about 40 km separates the islands from the mainland Tanzania (Fig 1). Mafia is an archipelago of islands, with the main island surrounded by seven small islets. Of these, five islets namely, Mafia (main islet), Bwejuu, Jibondo, Juani and Chole are inhabited. Mafia Islands have an estimated population of 50,167 people [28], of which 92.6% live in the main island of Mafia. The inhabitants of Mafia islands are subsistence farmers of coconuts and rice, and some are fishermen. The islets receive two rain seasons, long rains in March to June and short rains in October to December. As in many coastal areas of Tanzania, LF is an important mosquito borne diseases on the Mafia Islands. Before the start of MDA campaign in 2000, the baseline prevalence of *W*. *bancrofti* circulating filarial antigens (CFA) on the Mafia Islands was 49% and declined to 4% in 2006 [29]. This drop of prevalence indicates that the MDA rounds had marked impacts on the prevalence of *W*. *bancrofti* [30], but not yet reached the elimination threshold.

### Mosquito collection

Three villages, Kilindoni and Kiegeani (from the main Mafia Island) and Chole islet were purposely selected for the study. Other islets were excluded due to transport-related challenges and very low filarial vectors collected during preliminary surveys. All hamlets in Kiegeani and Chole villages (3 hamlets each) and an equal number of hamlets were selected from Kilindoni village in a non-random fashion to increase the odds of catching mosquitoes. All households in the selected hamlets were mapped using hand held Global Positioning System (GPS) device (Garmin *etrex* Legend H, Garmin ltd, USA). Three households in each hamlet were randomly selected for indoor mosquito collections using Centers for Disease Control (CDC) light traps (John W Hock Co, Gainesville, FL, USA). Light trapping was conducted as previously described [31] and mosquitoes were collected in each of the selected households every other day from 22^nd^ January to 10^th^ March 2014, resulting in a total of 22 light trap catch nights. In brief light traps were set in the evening between 17.00 to 18.00 hours and retrieved from 06.00 to 8:00 hours the following morning. Caught mosquitoes were transferred from the traps to labelled paper cups covered with netting material and transported to the field laboratory for identification and processing.

Moreover, two households were selected from each of the 9 study hamlets for outdoor mosquito collection using CDC gravid traps (John W. Hock Co., Gainesville FL). Gravid traps were set in peri-domestic areas and trapping was conducted as described previously [25, 32]. Traps were set in the evening between 17:00 and 18:00 hours, and retrieved the following morning between 06:00 and 08:00 hours in alternating days from 26^th^ January to 7^th^ March 2014. At each household the traps were ran for 18 nights. Collected mosquitoes were treated as described for light trap catch. Upon arrival in the field laboratory live mosquitoes were knocked down with chloroform and both (live and dead) were identified using morphological criteria [33,34]. In the field laboratory, freshly killed *Cx*. *quinquefasciatus*, *Anopheles gambiae* complex and *An*. *funestus* group were processed for *W*. *bancrofti* detection by microscopy. The rest were stored in Eppendorf tubes containing silica gel desiccants for later identification of sibling species of *An*. *gambiae* complex, *An*. *funestus* group and detection of *W*. *bancrofti* by PCR technique.

### Sibling species identification

Members of the *An*. *gambiae* complex were identified by PCR based on method previously described to identify *An*. *gambiae* sensu stricto (s.s), *An*. *arabiensis*, *An*. *quadriannulatus*, *An*. *melas* and *An*. *merus* [35, 36]. In brief, DNA was extracted by using Bender buffer method [36, 37] that involved homogenizing individual mosquito and precipitating extracted DNA using potassium acetate and ethanol. PCR reactions were conducted in a final volume of 20μl consisting of 0.25μM of each of the five primers, 1:1 TEMPase Hot Start polymerase master mix (Ampliqon III, VWR-Bie Berntsen, Denmark) and 2μl of DNA extract. The samples were amplified in GeneAmp PCR Systems 9700 (Applied Biosystems, USA) and cycling conditions were 95°C for 15 minutes followed by 35 cycles of denaturation at 94°C for 30 seconds, annealing at 50°C for 30 seconds, extension at 72°C for 30 seconds and final extension at 72°C for ten minutes.

On the other hand, sibling species of the *An*. *funestus* group were identified based on species-specific primers in the ITS2 region on the rDNA genes, a method previously described to identify *An*. *funestus*, *An*. *vaneedeni*, *An*. *rivulorum*, *An*. *leesoni* and *An*. *parensis* [38, 39]. DNA was extracted as described previously for sibling species of the *An*. *gambiae* complex. Each PCR run was conducted in a final volume of 25 μl consisting of 0.5 μM of each of the six primers, 1:1 TEMPase Hot Start polymerase master mix and 3 μl of DNA extract. The samples were amplified in GeneAmp PCR Systems 9700 and cycling conditions were 94°C for 15 minutes followed by 45 cycles of denaturation at 94°C for 30 seconds, annealing at 50°C for 30 seconds, extension at 72°C for 40 seconds and final extension at 72°C for ten minutes.

### Detection of *Wuchereria bancrofti*

Freshly killed *An*. *gambiae* s.l., *An*. *funestus* group and *Cx*. *quinquefasciatus* from both light and gravid traps were dissected and examined under microscopy for the first, second and human infective third stage larvae of *W*. *bancrofti* as previously described [40]. The required sample size for filarial vectors (mainly *Cx*. *quinquefasciatus*) examined by microscopy was estimated based on thresholds criteria outlined by the World Health Organization [41]. Moreover, using PCR technique, a separate sample (proportionally equal to dissected specimens) of randomly selected filarial mosquito vectors were pooled (25 mosquitoes in each reaction tube) and examined for presence of *W*. *bancrofti* infection as previously described [39, 42]. DNA was extracted from the pooled mosquitoes in the same way as explained for identification of sibling species of *An*. *gambiae* s.l. and *An*. *funestus* group. Extracted DNA was examined for presence of *W*. *bancrofti* by PCR targeting a highly repeated DNA sequences (the SspI repeat) found in *W*. *bancrofti*. In the reaction mixture, each of the 20 μl of PCR consisted of 0.25μM of each of the two primers (NV1&NV2), 1:1 Hot-Start TEMPase polymerase master mix and 2 μl of DNA extract. PCR thermal cycling conditions were 95°C for 15 minutes followed by 54°C for 5 minutes: then 35 cycles of denaturation at 94°C for 20 seconds, annealing at 54°C for 30 seconds, extension at 72°C for 30 seconds and final extension at 72°C for 5 minutes.

The amplified DNA for both sibling species and *W*. *bancrofti* specimens were separated based on their fragment size by gel electrophoresis and visualized under ultra violet light as previously described [35, 38].

### Data analysis

Data were entered in Excel and later transferred to STATA 12 (Stata Corp, College Station, Tx, USA) for analysis. The "infectivity rate" of the dissected mosquitoes was calculated as the percent of mosquitoes infected with infective larvae (L3) and the "infection rate" as the percent of mosquitoes infected with any stage of the parasite (L1, L2 and/or L3). For the PCR technique used for pooled mosquitoes, the probability that any one mosquito is infected with any stage of the *W*. *bancrofti* parasite were calculated using Poolscreen 2.02 software, providing maximum likelihood estimates for the rate of infection [43]. The 324 mosquito pools screened for *W*. *bancrofti* by PCR were randomly selected from a total of 563 pools made using random number generator programme in Microsoft Excel 2007. Mosquito infection and infectivity rates were compared using two sample test of proportions and p-value ≤ 0.05 was considered statistically significant.

### Ethical considerations

The study received ethical approval from the Medical Research Coordinating Committee of the National Institute for Medical Research, Tanzania (Ref: NIMR/HQ/R.8a/VOL. 9/1616). Before data collection, meetings were held with the district and respective village leaders to inform them about the study and to obtain their cooperation. Written informed consent was obtained from the heads of households before commencing mosquito collection in their respective houses or peri-domestic areas.

## Results

### Mosquito abundance and composition

A total of 38,505 mosquitoes were collected in the three villages of Chole, Kiegeani and Kilindoni during the study period. CDC light and gravid traps collected 17,831 (46.3%) and 20,674 (53.7%) mosquitoes, respectively. Out of the collected mosquitoes, 35,534 (92.3%) were filarial vectors belonging to members of the *An*. *gambiae* complex (1.3%), *An*. *funestus* group (0.5%) and *Cx*. *quinquefasciatus* (98.2%). All members of the *An*. *funestus* group and 99.8% of the members of *An*. *gambiae* complex were collected with light trap method. On the other hand, of 34,899 collected *Cx*. *quinquefasciatus*, 57.8% were collected using gravid traps. Majority (72.8%) of the filarial mosquito vectors were collected in Kiegeani village (Table 1).

**Table 1:** Mosquito abundance by taxa and collection method in three villages on Mafia Islands.

Mosquito taxa collected	Chole		Kiegeani		Kilindoni		Total collected (%)
	Light trap (%)	Gravid trap (%)	Light trap (%)	Gravid trap (%)	Light trap (%)	Gravid trap (%)	
*An*. *gambiae* complex	2 (0.3)	0 (0.0)	93 (0.7)	1 (0.0)	352 (8.3)	0 (0.0)	448 (1.2)
*An*. *funestus* group	0 (0.0)	0 (0.0)	9 (0.1)	0 (0.0)	178 (4.2)	0 (0.0)	187 (0.5)
*Cx*. *quinquefasciatus*	208 (35.6)	1516 (95.9)	11044 (85.1)	14715 (99.7)	3122 (73.2)	4294 (99.1)	34899 (90.6)
Other species^¥^	374 (64.0)	64 (4.1)	1837 (14.1)	47 (0.3)	612 (14.4)	37 (0.9)	2971 (7.7)
Total by trap/village	584	1580	12983	14763	4264	4331	38505

^¥^Other non-filarial vector mosquitoes

Of the collected Anopheles, 270 members *An*. *gambiae* complex and 114 *An*. *funestus* group were processed for sibling species identity using PCR technique. *An*. *gambiae* sensu stricto (s.s) sibling species accounted for 88.3% of the analysed members of the *An*. *gambiae* complex. Other members of the *An*. *gambiae* complex identified were *An*. *arabiensis*, *An*. *quadriannulatus* and *An*. *merus*. On the other hand, *An*. *funestus* s.s was the majority (99.1%) of the identified sibling species in the *An*. *funestus* group (Table 2).

**Table 2:** PCR identification of sibling species of the *An*. *gambiae* complex and *An*. *funestus* group.

Species complex	No. included in test	No. of positive PCR test	Sibling species identified	Total No. (%)*
*An*. *gambiae* complex	270	265**	*An*. *gambiae* s.s.	234 (88.3)
			*An*. *quadriannulatus*	16 (6.0)
			*An*. *arabiensis*	13 (4.9)
			*An*. *merus*	2 (0.8)
*An*. *funestus* group	114	111^μ^	*An*. *funestus* s.s.	110 (99.1)
			*An*. *leesoni*	1 (0.9)

* = Percent of the positive PCR test;

** = PCR negative specimens were not processed further

### Vector infection and infectivity with *W*. *bancrofti*

A total of 3,866 filarial mosquito vectors collected with CDC light traps were dissected and examined for infection and infectivity with *W*. *bancrofti*. Nine (0.23%) *Cx*. *quinquefasciatus* were found to be infected with any of the three larval stages (L1, L2 and /or L3) of *W*. *bancrofti* and three mosquitoes (0.08%) were infective. None of the dissected members of the *An*. *gambiae* s.l. and *An*. *funestus* were found to carry *W*. *bancrofti* larvae of any stage. On the other hand, a total of 4,070 *Cx*. *quinquefasciatus* mosquitoes collected with CDC gravid trap were dissected and examined for infection and infectivity with *W*. *bancrofti*. Eleven (0.27%) *Cx*. *quinquefasciatus* were found to be infected with any of the three larval stages (L1, L2 and /or L3) of *W*. *bancrofti* and three (0.07%) were infective. Mosquito infection and infectivity rates between the two trap types were not significantly different (Table 3).

**Table 3:** Comparison of filarial vectors infection and or/infectivity rate as measured by microscopy and PCR.

Method of analysis	Trap type	No. analysed	No. infected (%)	P-value*	No. infective (%)	P-value**
Microscopy	Light trap	3866^¥^	9 (0.23)	-	3 (0.08)	-
	Gravid trap	4070	11 (0.27)	P = 0.7393	3 (0.07)	P = 0.9498
	All traps	7936	20 (0.25)	-	6 (0.08)	
PCR	Light trap	161^£^†	45 (1.3)ǂ	-	-	-
	Gravid trap	163†	70 (2.2)ǂ	P> 0.05	-	-
	All traps	324†	115 (1.7)ǂ	-	-	-

^†^Pools of 25 mosquitoes each;

^ǂ^ Infection rate (Pools Screen V2.0.2; Likelihood ratio method; 95% CI for all traps (1.4–2.1), gravid trap (1.7–2.9) and light trap (0.9–1.8);

*Two sample test of proportions comparing infection rate between the trap types;

**Two sample test of proportions comparing infectivity rate between the trap types;

^¥^Filarial vector composition: *Cx*. *quinquefasciatus* = 3767; *An*. *gambiae* complex = 73 and *An*. *funestus* group = 26;

^£^Filarial vector pools: *Cx*. *quinquefasciatus* = 155; *An*. *gambiae* complex = 4 and *An*. *funestus* group = 2

Using PCR technique, of 324 mosquito pools (each with 25 mosquitoes) tested, 115 were found to be infected with at least a larval stage of *W*. *bancrofti*. Analysis by trap type revealed that of 163 gravid trap mosquito pools processed, 70 were infected whilst out of 161 light trap pools processed, 45 pools were infected. The infection rates between the two trapping methods were not significantly different (two sample test of proportions, p>0.05). On the other hand, of 6 *Anopheles* pools processed, only one (belonging to *An*. *funestus* group) was infected. For both trap types and species, the probability that any one mosquito in the pool was infected with any stage of the *W*. *bancrofti* parasite was estimated at 1.7%. Comparison of mosquito infection rates as measured by the two xenomonitoring methods have shown that PCR estimate seven-fold higher infection rate than dissection (Table 3).

## Discussion

Mosquitoes belonging to the *An*. *gambiae* s.l., *An*. *funestus* and *Cx quinquefasciatus* are the vectors of *Wuchereria bancrofti* in Tanzania as well as in many other parts of Sub-Saharan Africa [3, 40, 44, 45]. In their review, Bockarie and colleagues [3] examined the potential role of vector control as a supplementary component of MDA based strategies in LF elimination in different epidemiological settings. Inclusion of vector control was predicted to lower the number of MDA cycles even in areas with less than optimal treatment coverage [3]. Of particular relevance, inclusion of vector control has been considered crucial in LF elimination where *Culex* and *Aedes* mosquitoes are involved in the transmission [3].

Studies have documented an increased potential of *Cx*. *quinquefasciatus* as vector due to its expanding population and its inherent efficiency in LF transmission as the prevalence of the disease falls [3, 46, 47]. The current study searched for potential LF vectors on Mafia Islands in an attempt to validate and deploy vector control method based on "lure and kill" [48] to accelerate LF elimination efforts.

Previous studies in north eastern Tanzania documented principal vectors of *W*. *bancrofti* in order of decreasing importance to be members of *An*. *funestus* group, *An*. *gambiae* complex and *Cx*. *quinquefasciatus* [40, 44, 49, 50]. In the current study, *Cx*. *quinquefasciatus* accounted for 98.2% of the filarial mosquito vectors caught and *W*. *bancrofti* infection and infectivity was confined to this vector. The findings of the current study are supported by studies conducted recently in north-eastern Tanzania indicating a shift in filarial vector from transmission by anophelines to *Cx*. *quinquefasciatus* [19, 25]. *Cx*. *quinquefasciatus* has been described to be the predominant vector of lymphatic filariasis in urban areas of the neighbouring Islands of Zanzibar [51]. Previously, *Cx*. *quinquefasciatus* was considered an urban vector but it has become successful in establishing itself in the rural areas possibly due to adoption of urban life in rural areas [46,47]. It is likely that *Cx*. *quinquefasciatus* will remain an important LF vector in many parts of coastal Tanzania following the reported decline in anopheline vectors [45].

In mosquito surveys, CDC light trap has been considered as an important tool as it collects a proportion of mosquitoes involved in the transmission (host seeking) and compares fairly well with a standard method based on human landing catch [52]. On the other hand, light traps do collect both anopheline and culicine mosquitoes, both of which are filarial vectors [25, 44, 45]. Other studies have suggested that by collecting population of mosquitoes that have taken at least one blood meal, gravid traps are ideal for xenomonitoring [53,54]. However, a study comparing the CDC light and gravid traps as tool for xenomonitoring concluded that gravid traps may be useful where *Cx*. *quinquefasciatus* is the only vector [25]. The current study has shown that *W*. *bancrofti* infection and infectivity rates detected by microscopy from mosquitoes collected with CDC light and gravid traps were not significantly different. However, due to the fact that, the former target host seeking while the later collect preferentially gravid *Cx*. *quinquefasciatus*, the use of any trap type should be adapted to the prevailing local LF vectors. Based on our findings, in areas where *Cx*. *quinquefasciatus* is the main vector, either of the traps is likely to provide accurate information on ongoing LF transmission in xenomonitoring. However, in areas where LF is anopheline transmitted, or both vectors prevail, light trap is an ideal tool for xenomonitoring [25].

Dissection of vectors to detect *W*. *bancrofti* infection in mosquitoes has been considered a gold standard method for LF xenomonitoring [19, 40]. However, studies have shown that, as the prevalence of LF decrease following repeated rounds of MDA, molecular based technique with high throughput and precision are ideal for xenomonitoring [26, 27]. The findings of the current study have shown that PCR was able to detect more filarial infection in mosquitoes compared to dissection. It was moreover evident that *W*. *bancrofti* infection rates detected by PCR form mosquitoes collected by CDC light and gravid traps were not significantly different. However, it should be noted that *W*. *bancrofti* parasites detected by PCR in the mosquitoes included all the vector-borne stages, since the PCR test used was not designed to discriminate between infective and non-infective stages of the parasite.

While detection of infected mosquitoes is an indication of the existence of a reservoir of microfilaraemia in human population, presence of infective mosquitoes harbouring L3 stages of *W*. *bancrofti* signifies ongoing transmission. The findings of this study provide an indication of potential on-going transmission of *W*. *bancrofti* on Mafia Islands. In a neighbouring district of Rufiji, the prevalence of *W*. *bancrofti* circulating filarial antigens (CFA) among schoolchildren was recently reported at 14.4% suggesting that transmission of LF has continued in the area despite nine rounds of MDA [30]. Mosquito infectivity rate reported in the current study was lower than that reported prior to start of MDA campaign in Tanzania [19, 40] and comparable to the situation after 5 rounds of MDA in north-eastern Tanzania [45]. It was evident that *Cx*. *quinquefasciatus* was the main filarial vector on Mafia Islands, that worth to be targeted with a vector control intervention as supplement to the ongoing MDA to accelerate LF elimination efforts. With these findings, there is an urgent need to assess the extent of on-going transmission through doing a follow-up survey on people including the use of the novel antibody test for *W*. *bancrofti* L3 larvae antigens [55].

### Conclusions

The study has shown that *Cx*. *quinquefasciatus* was the dominant man-biting mosquito on Mafia Islands and *W*. *bancrofti* infection is confined to this vector group. Both CDC light and gravid traps were found useful for mosquito vector surveillance. Moreover, it was found out that molecular method based on PCR was seven fold more sensitive than dissection in detecting *W*. *bancrofti* infection in mosquitoes. By using xenomonitoring as proxy to human infection, the study indicated that *W*. *bancrofti* transmission was still ongoing on Mafia Islands after more than a decade of control activities based on MDA. Our findings suggest that inclusion of mosquito control method that target *Cx*. *quinquefasciatus* will accelerate LF elimination on Mafia Islands and other coastal areas of Tanzania.
